# FOUR TYPES OF FINANCIAL SUPPORT AMONG “SANDWICHED” MIDDLE-AGED ADULTS: LIFE AND FAMILY RELATIONSHIP SATISFACTION

**DOI:** 10.1093/geroni/igae098.4194

**Published:** 2024-12-31

**Authors:** Hyeonji Cho, Meejung Chin, Catherine García

**Affiliations:** Syracuse University, Syracuse, New York, United States; Seoul National University, Seoul, Republic of Korea; Syracuse University, Syracuse, New York, United States

## Abstract

Middle-aged adults who provide supports to both their parents and children – often referred to as the “sandwiched generation” – are at higher risk for poor mental health. However, within the Korean cultural context, where strong expectations for intergenerational financial support exists, may influence the well-being of individual’s who either fulfill or fail to meet these expectations. This study classified middle-aged adults into four groups based on their financial support patterns: (a) Supporting both parents and children, (b) supporting only parents, (c) supporting only children, and (d) Supporting neither. Using data from the 25th wave (2022) of the Korean Labor and Income Panel Study (KLIPS), we analyzed 1,975 middle-aged householders aged 40-64, all of whom have at least one living parent and co-reside with at least one child ([a]=72.6%, [b]=14.2%, [c]=9.8%, [d]=3.4%). Controlling for sociodemographic and economic characteristics, we conducted linear regression analyses to examine the association between financial support groups (group [a] as the base level) and life satisfaction indicators (i.e. overall life satisfaction and family relationship satisfaction). Regarding overall life satisfaction, group (a) was more satisfied than group (b) (B=-0.09, p=.031) and (c) (B=-0.16, p<.001). Additionally, group (a) was more satisfied in their family relations than group (c) (B=-0.14, p=.002) and (d) (B=-0.14, p=.046). These results suggest that, in Korea, the societal expectation to fulfill financial responsibilities towards parents may contribute to a greater sense of life satisfaction among those who successfully meet these obligations, whereas those unable to do so might experience diminished well-being.

